# VRAM Flap for Pelvic Floor Reconstruction after Pelvic Exenteration and Abdominoperineal Excision

**DOI:** 10.3390/jpm13121711

**Published:** 2023-12-14

**Authors:** Ionut Flaviu Faur, Adelina Clim, Amadeus Dobrescu, Catalin Prodan, Rami Hajjar, Paul Pasca, Marco Capitanio, Cristi Tarta, Alexandru Isaic, George Noditi, Ionel Nati, Bogdan Totolici, Ciprian Duta, Gabriel Lazar

**Affiliations:** 1 IInd Surgery Clinic, Timisoara Emergency County Hospital, 300723 Timisoara, Romania; flaviu.faur@umft.ro (I.F.F.); catalin.prodan-barbulescu@umft.ro (C.P.); rami_hajjar@yahoo.com (R.H.); drpascapaul@gmail.com (P.P.); capitanio.marco@umft.ro (M.C.); tarta.cristi@umft.ro (C.T.); isaicus@gmail.com (A.I.); george.noditi@gmail.com (G.N.); ciprian_duta@yahoo.com (C.D.); 2 X Department of General Surgery, ”Victor Babes” University of Medicine and Pharmacy, 300041 Timisoara, Romania; 3 IInd Obstetric and Gynecology Clinic “Dominic Stanca”, 400124 Cluj-Napoca, Romania; clim.adelina@yahoo.com; 4 2nd Department of Obstetric & Ginecology, ”Iuliu Hatieganu” University of Medicine and Pharmacy, Victor Babeș Street Number 8, 400347 Cluj-Napoca, Romania; nati.ionel@yahoo.com (I.N.); dr.lazar.gabriel@gmail.com (G.L.); 5 Ist Clinic of Oncological Surgery, Oncological Institute “Prof. Dr. I. Chiricuta”, 400015 Cluj-Napoca, Romania; 6 Ist Clinic of General Surgery, Arad County Emergency Clinical Hospital, 310158 Arad, Romania; totolici_bogdan@yahoo.com; 7 Department of General Surgery, Faculty of Medicine, “Vasile Goldiș” Western University of Arad, 310025 Arad, Romania

**Keywords:** vertical rectus abdominis muscle flap, perineal wound, pelvic exenteration, squamosal cell carcinoma of the uterine cervix, reconstruction

## Abstract

Due to the still large number of patients diagnosed with pelvic neoplasms (colorectal, gynecological, and urological) in advanced stages right from the initial diagnosis, surgery represents the mainstay of treatment, often implying wide, eventually multi-organ resections in order to achieve negative surgical margins. Perineal wound morbidity, particularly in extralevator abominoperineal excision, leads to complications and local infection rates of up to 40%. Strategies to reduce postoperative wound complications are being pursued to address this issue. The VRAM flap remains the gold standard for autologous reconstruction after pelvic oncological resection; it was initially designed for abdominal wall defects and later expanded for large pelvic tissue defects. The flap’s application is based on its physical characteristics, including abundant tissue and a generous skin paddle, which effectively obliterates dead space after exenterations. The generous skin paddle offers good cosmetic and functional outcomes at the recipient site. This article describes the case of a patient histopathologically diagnosed with stage IIIA squamous cell carcinoma of the uterine cervix who received multimodal onco-surgical treatment. The surgical mainstay of this treatment is pelvic exenteration. Pelvic reconstruction after this major surgery was performed using a vertical flap with the rectus abdominis.

## 1. Introduction

Cancer still represents a major cause of morbidity and mortality worldwide, regardless of socioeconomic characteristics and/or demographics. According to the GLOBOCAN stats published for 2020, an estimated 19.3 million new cancer cases and almost 10.0 million cancer deaths occurred [1,2]. Malignant tumors arising from the pelvic region seem to particularly contribute to the numbers mentioned above. Pelvic malignancy death rates are decreasing due to increased screening rates and global sensibilization campaigns, reducing financial burdens and promoting better health outcomes. Nonetheless, patients often present with advanced stages due to the lack of early and specific symptoms, the reason why they do not seek medical attention until signs of extensive loco-regional involvement appear [3]. 

Advanced or recurrent pelvic cancers can cause severe pain, bleeding, sepsis, obstruction, and fistula forms as symptoms. A few of the symptoms have also been linked to radiation treatment in the past. Primary and recurrent locally advanced carcinomas are the main indications for pelvic exenteration. The adjacent pelvic organs or surrounding anatomy, such as the pelvic sidewall, neurovascular structures, or the pelvis’ bony sacrum or pubis, are affected by these disorders. The objective is to accomplish a full oncologic (R0) resection, which is denoted by the absence of cancer in the resection margins [3,4,5].

The primary contraindication for pelvic exenteration is the inability to achieve clear surgical margins free of malignancy (R0). There is a generally accepted unwritten consensus that exenteration should only be offered with resectable disease and with curative intent due to the potential postoperative morbidity that may accompany pelvic extenteration [3]. 

When such stages of advancement are reached, surgery represents the mainstay of treatment, often implying wide, eventually multi-organ resections in order to achieve negative surgical margins [3,4,6]. 

R0 resection represents the main goal of such procedures, as it has been proven to chiefly influence and consistently predict disease-free survival and postoperative recurrence rates. Pelvic exenteration and abdominoperineal resection are the main procedures for locally advanced malignancies in the anorectum, gynecological, or urological pelvic organs [3,4,6]. 

Postoperative complications following pelvic exenteration can be classified into acute (immediate) and chronic (late) types. Acute local complications include massive bleeding, intestinal obstruction, skin flap necrosis, haematoma, intestinal fistula, urinary fistula, wound infection, peritonitis, pelvic abscess, stoma separation, ureteral obstruction, uraemia, stoma stenosis, prolonged ileus, pelvic cellulitis, perineal evisceration, stoma hernia, co-lostomy necrosis, loop necrosis, and arterial thrombosis. Potential acute systemic complications include renal, cardiovascular, neurological, respiratory, and metabolic issues. Late postoperative complications include intestinal obstruction, small bowel ileus, kidney stones, stoma hernia, and metabolic disorders [3,4,5,6].

The main source of morbidity is the perineal wound and complications, with an estimated rate of local infection of up to 40%. Flap reconstruction techniques for pelvic defects have gained popularity due to their potential to address surgical challenges and reduce infection and dehiscence risks by appointing healthy, vascularized autologous tissue [4].

The aim of this paper is to reveal the benefits of a perineal reconstruction technique using a vertical rectus abdominis flap after pelvic exenteration. The present case is an example of the reconstruction of the perineal anatomical region by means of a vertical rectus abdominis muscle flap, reducing the well-known complications of perineal lacerations after pelvic exenteration.

## 2. Materials and Methods

### Diagnostic and Stadialisation

A 63-year-old female without other associated pathologies presented at the Gynecology Department in April 2017 with metrorrhagia, faintness, and dizziness. 

Following clinical and paraclinical examination, the diagnosis of menopausal metrrhoragia was established and a primary carcinoma of the cervix was suspected. Aside from the levels of red blood cell (RBC) and hemoglobin (HGB) of 4.01 and 7.1, respectively, the patient’s laboratory results were normal. 

A biopsy of the cervix was performed and the excised material was sent for histopathological examination. Histopathological results: squamous cell carcinoma of the cervix—G1 stage IIIA.

This CT scan of the thorax, abdomen, and pelvis (15 June 2017). showed rare solid nodules (1–2/pulmonary field); the cervix enlarged in dimensions with a transverse diameter of 5 cm, due to a proliferative process with an infiltrative appearance of approximately 4 cm in diameter, also extended to the level of the uterine body. The tumor came into contact with the posterior wall of the urinary bladder and the anterior wall of the rectum, which seemed to be clearly defined. Infiltration of both parameters and minimal carcinomatous infiltration of pelvic fat showed fine solid septa and rare hyperdense micronodules. Rare infracentimeter lumboaortic adenopathies.

The MRI of the abdomen + pelvis (18 October 2022) showed a tumor formation at the level of the cervix, 7.8 cm, circumferential, with bilateral parametrial invasion and lower extension at the level of the vagina and vulva on the right side, invasion of the levator ani muscle on the right side, with total invasion of the urethra along the entire path, and in contact with the anterior rectal wall without MRI signs of invasion, and multiple bilateral enlarged inguinal lymph nodes up to 14 mm in size without secondary dissemination at the level of the scanned bone structures.

CTs of the thorax + abdomen + pelvis (31 October 2022) were performed due to the patient’s enlarged inguinal lymph nodes. In the region of the cervix, a formation of approximately 55/40 mm diameter with a hypodense center and iodophilic, irregular periphery is evident. Without suspicious pelvic adenopathies, retroperitoneal lymph nodes of 6–7 mm, and without signs of bone metastases.

The PET-CT scan (December 2022) confirmed the presence of a 75/45 mm FDG (SUVlbm = 7.9) hypercapturing tumor formation centered on the cervix with invasion in the vagina up to the perineal level, without significant dimensional or metabolic active retroperitoneal, pelvic, or inguinal adenopathy. 

## 3. Results

### 3.1. Treatment Plan

During the patient’s hospitalization, hemostatic and analgesic treatment was administered (Alindor 500 mg, Adrenostazin 0.3 mg/mL, Etamsylate 250 mg, Fitomenadion 10 mg/mL). The patient was discharged with the recommendation of a gynecological consultation in 2 weeks. 

External radiotherapy with intensity radiated modulation therapy (IMRT) for neoadjuvant purposes was proposed (06–07/2017). The patient was recommended to undergo radio-chemotherapy sessions with brachytherapy. In June and July 2017, radiotherapy was practiced for neoadjuvant purposes up to a total dose of 42 Gy/ 21 fr. (which included the pelvic nodules, uterus, both parameters, and the upper third of the vagina). Chemotherapy was also proposed, but unfortunately, the patient refused it.

Cancellation of brachytherapy (17 July 2017).

It should be mentioned that brachytherapy could no longer be performed due to hemorrhage (local hemostatic treatment with Gelaspon and intravenous Adrenostazin and Etamsylate was administered).

The patient was indicated for radical oncological surgery—pelvic exenteration.

### 3.2. Expected Outcome of the Treatment Plan

Evolution without treatment (disease progression without specialized therapy).

Without treatment, cervical cancer progresses more slowly or more rapidly towards loco-regional invasion (of regional lymph nodes, the parametrium, nearby blood vessels and neighboring organs) and systemic metastases (lung, bone, liver, distant lymph nodes).

Evolution with specialized treatment.

This category details the evolution and possible complications following surgical, radiotherapy and chemotherapy treatment.

Total pelvic exenteration has been used as a salvage therapy. Candidates are those who have failed radiation therapy or primary surgical or combined treatment of the recurrence in the central pelvis and in cases of locally advanced primary pelvic tumors.

### 3.3. Actual Outcome

In October 2022, the patient who was treated with exclusive irradiation in 2017, presented with pelvic recurrence with the invasion of the vagina, urethra, and parameters bilaterally. 

Clinical examination revealed the presence of a friable, hemorrhagic tumor formation at the level of the right hemivulva and vagina.

### 3.4. Follow-Up

Post-therapeuthic follow-up was performed by successive clinical consultations and imaging examinations at 3, 6, 12 months postoperatively. The patient was declared disease free.

### 3.5. Literature Review

A brief advanced research on PubMed about the topic—carried out using the MeSH terms “perineum” and “surgical flaps”—yielded a total of 14 results by filtering the level of the study to only meta-analyses and systematic reviews. Further on, studies addressing either primary benign or secondary pathology, such as local infection (either primary or secondary after previous pelvic surgery), benign proctology, hidradenitis suppurativa, were excluded, leaving a total pool of 6 contextually relevant studies—they are listed in Table 1. All of them seem to universally agree by proof of statistical evidence on how flap reconstruction procedures positively influence outcomes and wound healing compared to primary closure techniques [7,8,9,10] even though the marked heterogenicity of the various study populations in terms of the different types of flaps that were adopted does not allow for a better insight on the VRAM flap itself. While Johnstone et al. in 2017 managed to perform a statistical analysis that proved how the VRAM technique presents a lower incidence of perineal wound and flap complications [11], Eseme et al. in 2022 gathered data from 925 patients in a proportional meta-analysis strictly comparing the VRAM flap with the gracilis one, recording a higher incidence of donor site hernias [12]. Mean follow-up was not reported in all the studies mentioned above, while other patient data potentially crucial for the reliability of study outcomes was absent or heterogeneously expressed (e.g., mean age, prevalence of neoadjuvant treatment). Standardization probably represents the key factor for information reporting in order to establish some clear indications meant to aid the surgeon through the decision making process in the best interest of patient prognosis. A noteworthy insight was already proposed by Yan et al. into a meta-analysis that evaluated the results obtained through the application of enhanced recovery after surgery (ERAS) protocols in the postoperative management of autologous flap-based reconstruction, proving a substantial benefit manifested by decreased length of hospital stay without observing an increase in the incidence of complications [13,14]. Despite that, the lack of a strong, statistically uniform body of data clearly supporting such protocols and measures for enhanced recovery in the field of flap-based perineal reconstruction does not allow for their standard applications on a global scale; further research is therefore still warranted.

### 3.6. Surgical Intervention

**Figure 1:**
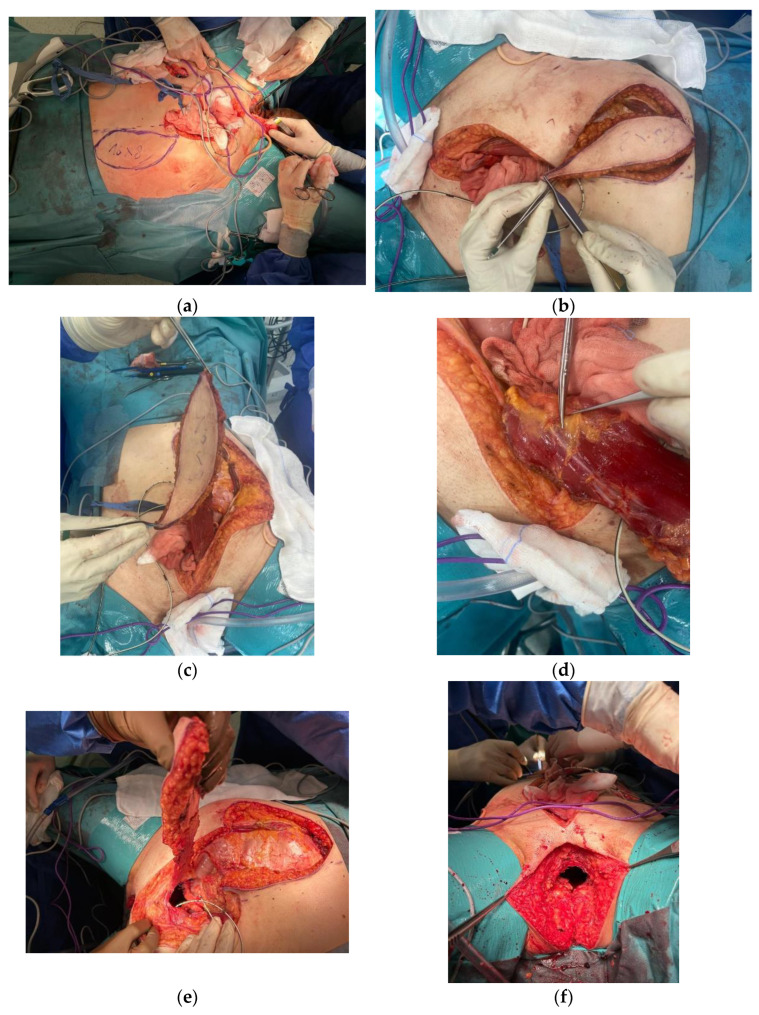

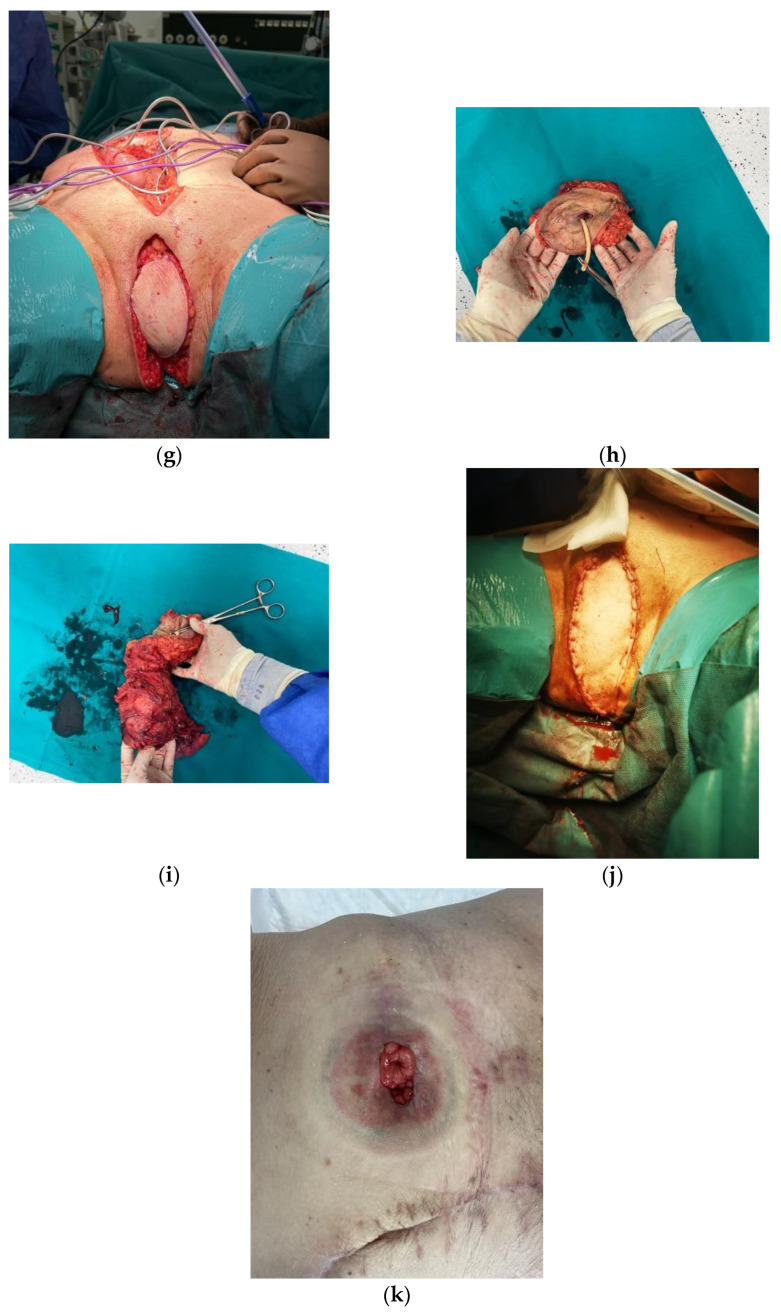
VRAM Flap for Pelvic Floor Reconstruction after Pelvic Exenteration. (**a**) Pre-incision measurement of the desired/ intended abdominal myocutaneous flap (16 × 8 cm). (**b**) Preparation of the myocutaneous flap of the rectus abdominis muscle. (**c**–**e**) Preparation of the myocutaneous flap of the rectus abdominis muscle. (**f**,**g**) Appearance of the perineal wound before and after the application of the flap. (**h**,**i**) En-bloc surgical resection specimen (**j**) Final appearance of the perineal region after vertical abdominal rectus muscle flap reconstruction. (**k**) Final aspect of the stoma and of the abdominal scar.

The technique shown in Figure 1 has been used for 4 years, with an average of 20 surgical procedures (VRAM reconstruction after pelvic exenteration) per year within this surgical team.

## 4. Discussion

Malignant tumors arising from the pelvic region seem to particularly contribute to the numbers mentioned above. Declining death rates for pelvic malignancies such as colorectal cancer (CRC), prostate cancer, cervical cancer, and other gynecological entities can be substantially ascribed to the increased rate of screening observed in recent years as a result of intensive sensibilization campaigns deployed by governments around the world in order to buffer the financial burden that cancer exerts as an entity. Nonetheless, patients often present with advanced stages due to the lack of early and specific symptoms, the reason why they do not seek medical attention until signs of extensive loco-regional involvement appear. 

Pelvic exenteration (PE), initially described by Brunschwig in 1948 in New York as a palliative procedure for recurrent carcinoma of the cervix [6], together with abdominoperineal resection (APR), firstly proposed by Miles in 1908 [5] but subsequently revised and modified [16], are the two main procedures that are currently performed for locally advanced malignancies arising from either the anorectum, gynecological, or urological pelvic organs. What probably represents the main source of morbidity, especially in the case of extralevator abominoperineal excision (ELAPE), is the perineal wound and its complications, with an estimated rate of local infection up to 40%. Other complications are not uncommon: from early fluid collections, which eventually turn into abscesses, and wound dehiscence, to chronic fistulas and perineal hernias due to weakening of the pelvic floor; also, the migration of bowel loops into the large remaining pelvic defect has been observed, leading to bowel movement disturbances and a reduction in the patient’s quality of life. Techniques using the omentum to suspend the bowel and ileal conduit out of the pelvis or fill it have reduced the incidence rate for abscesses and fistulas [17].

Due to such levels of perineal wound morbidity, coupled with the steadily increasing number of pelvic exenteration and ELAPE procedures that are performed, different strategies have been pursued in order to try and lower the incidence of postoperative wound complications. Primary closure of the wound probably revealed itself as the riskier option available, especially considering the increasing prevalence of patients presenting for surgery after neoadjuvant radiotherapy, which significantly impacts pelvic tissues, making them prone to develop local ischemia, fibrosis, and poor healing potential—some series report complication rates up to 57% [18]. Moreover, as already mentioned above, primary closure harbors a greater risk for bowel herniation into the pelvic space; this issue has also been addressed by the introduction of biological mesh repair techniques that reduce tissue tension inside the wound: so far they have been widely employed around the world with satisfactory results and lower rates of perineal hernia compared to primary closure. Unfortunately, despite lowering tension, biological meshes do not bring benefits regarding other common points of concern encountered along the healing phase of the postoperative perineal wound, such as the tendency for fluid collection with consequent early seromas and abscesses, and delayed healing due to the surrounding, poorly vascularized tissues affected by neoadjuvant radiotherapy [19]. 

Flap reconstruction techniques for pelvic defects gained growing popularity over the years, most likely because of their potential to aid facing the main surgical challenges presented by the rising prevalence of irradiated patients; in fact, by the apposition of new, healthy and vascularized autologous tissue, not only do local tension levels decrease, but so does the infection and dehiscence risk due to the greater supply of oxygen, nutrients, and inflammatory cells to the wound bed [20]. 

In terms of common practice, the current gold standard for autologous reconstruction after pelvic oncological resection remains the vertical rectus abdominis muscle (VRAM) flap, first described by Mathes and Botswick in 1977 for the repair of various defects of the abdominal wall, and further adapted for large pelvic tissue defects [21,22]. The rationale behind its use relies on some critical crucial factors: first, the physical characteristics of the flap itself, provided with abundant amounts of tissue forming a bulky, optimally vascularized mass well suited for obliterating the dead space left after large exenterations; second, the generous skin paddle provides good cosmetic and functional outcomes at the recipient site; and last but not least, the anatomical features of the pedicle mobilized for this flap allow for a good range of movement and rotation to reach distant defects [23,24]. 

Other options for flap closure are represented by inner thigh flaps, with particular attention reserved for the gracilis flap [24], requiring lower expertise for tailoring and preparation and supposedly harboring a lower risk for donor site complications, especially ventral hernias, compared to VRAM. The choice for the perfect technique for pelvic reconstruction fuels many debates worldwide in terms of comparison between the various procedures and their individual indications and/or advantages, especially due to the various discrepancies between findings published in the literature and the lack of large studies with low interpretation biases.

## 5. Conclusions

The rectus abdominis muscle flap could eventually eclipse all other techniques for the reconstruction of the pelvic floor after abdominoperineal resection and pelvic exenteration. 

According to the narrative review and the case presented, the “vertical rectus abdominis muscle—VRAM” technique shows the best aspects of postoperative morbidity and mortality, so it could become a gold standard technique. Regarding the learning curve, in our experience, a multidisciplinary team is needed and the minimum number of cases to reach this learning curve is 30 surgeries as the main author.

Multi-center studies are required in this context to collect a significant amount of data indicating and proving the utility of the rectus abdominis flap above and beyond the other flap techniques that are already commonly used and discussed in the medical literature.

## Figures and Tables

**Table 1:** Meta-Analysis and systematic reviews identified after advanced research on PubMed (MeSH terms “perineum”, “surgical flaps”) and exclusion. * = expressed as a mean.

Reference	Study Design	Total No. of Patients	Mean Age	Type of Cancer	Type of Resection	Type of Flap	Duration of Follow-Up	Conclusion
Devulapalli et al. 2016 [8]	Meta-Analysis	566	59.1	Rectal Cancer, Anal Cancer, Others	APE, PE	VRAM, Gracilis	24 months *	Flap reconstruction reduces wound morbidity after APE, PE compared to primary closure
Yang et al. 2019 [12]	Meta-Analysis	17.913	Not specified	Rectal Cancer	APE, ELAPE, PE	VRAM, Gracilis, Gluteal	17–50.4 months	Flap reconstruction reduces wound complication compared to primary closure
Eseme et al. 2022 [9]	Proportional Meta-Analysis	925	50.3–67	Rectal, Anal, Vaginal, Others	APE, PE	VRAM, Gracilis	NS	Flaps are safe for vulvoperineal reconstruction; VRAM raises risk for donor site hernia
Buscail et al. 2021 [10]	Meta-Analysis	2180	Not specified	Rectal, Anal, Others	APE, PE	VRAM	NS	Flaps reduce incidence of wound complication after APE
Johnstone, 2017 [11]	Systematic Review	649	Not specified	Rectal, Anal, Gynecological, Others	APE, PE	VRAM, Gracilis, Gluteal	NS	VRAM superior in terms of incidence of wound complication
Foster et al. 2012 [15]	Systematic Review	522 (one study NS)	Not specified	Rectal, Anal	ELAPE	VRAM, Gracilis, Gluteal,	10–38 months *	No significant difference in complications between flap and mesh repairs

## Data Availability

Data are contained within the article.
